# Knowledge, experiences, and attitudes of Australian General Practitioners towards medicinal cannabis: a 2021–2022 survey

**DOI:** 10.1186/s12875-022-01946-x

**Published:** 2022-12-19

**Authors:** Zeeta Bawa, Danielle McCartney, Ramesh Manocha, Iain S. McGregor

**Affiliations:** 1 The University of Sydney, Lambert Initiative for Cannabinoid Therapeutics, Sydney, NSW Australia; 2 Brain and Mind Centre, The University of Sydney, Sydney, NSW Australia; 3 Faculty of Science, School of Psychology, The University of Sydney, Sydney, NSW Australia; 4 Sydney Pharmacy School, The University of Sydney, Sydney, NSW Australia; 5 Healthed, Sydney, NSW Australia

**Keywords:** Cannabis, Cannabinoid, Medicinal cannabis, General practice, Doctor

## Abstract

**Background:**

Medicinal cannabis (MC) products have been available on prescription in Australia for around six years. General practitioners (GPs) are at the forefront of MC prescribing and recent years have seen substantial increases in prescription numbers. This study examined the current knowledge, experiences, and attitudes of Australian GPs around MC. We also compared our findings to those of an earlier 2017 investigation.

**Method:**

We conducted a cross-sectional study using a 42-item on-line questionnaire adapted from our earlier 2017 survey. The current survey was completed by GPs attending an on-line, multi-topic educational seminar. Australian GPs (*n* = 505) completed the survey between November 2021 and February 2022. Data were synthesised using descriptive statistics. MC ‘prescribers’ and ‘non-prescribers’ responses were compared using Pearson’s χ2 tests.

**Results:**

While most GPs (85.3%) had received patient enquiries about MC during the last three months, only half (52.3%) felt comfortable discussing MC with patients. Around one fifth (21.8%) had prescribed a MC product. GPs strongly supported MC prescribing for palliative care, cancer pain, chemotherapy-induced nausea and vomiting, and epilepsy, more so than in our 2017 survey. Prescribing for mental health conditions (e.g., depression, anxiety) and insomnia received less support. Opioids, benzodiazepines, and chemotherapy drugs were rated as more hazardous than MC. GPs correctly endorsed concerns around Δ^9^-tetrahydrocannabinol-related driving impairment and drug-seeking behaviour. However, additional concerns endorsed around cannabidiol causing addiction and driving impairment do not agree with current evidence. Consistent with this, many GPs (66.9%) felt they had inadequate knowledge of MC.

**Conclusion:**

Acceptance of MC as a treatment option has increased among Australian GPs since 2017. However, there is a clear need for improved training and education of GPs around cannabis-based medicines to provide increased numbers of skilled prescribers in the community.

**Supplementary Information:**

The online version contains supplementary material available at 10.1186/s12875-022-01946-x.

## Background

The Australian Federal Government legalised medicinal cannabis (MC) in November 2016 [1, 2]. This has enabled patients to be prescribed a wide range of products containing Δ^9^-tetrahydrocannabinol (THC) and/or cannabidiol (CBD) [3, 4]. These products are mostly classified as ‘unregistered medicines’ by the Australian drug regulator (the Therapeutic Goods Administration, TGA) and doctors must apply for approval to prescribe such products through the Special Access Schemes (SAS-A or SAS-B) or the Authorised Prescriber (AP) Scheme [2, 3, 5]. The SAS schemes allows doctors to seek approval from the TGA to prescribe MC products to an individual patient, while the AP scheme provides permission to prescribe a MC product to multiple patients suffering from the same condition [6]. Both schemes place the onus of assessing clinical need, justifying MC prescribing, and choosing an appropriate MC product on the prescribing clinician. The subsequent prescription is then dispensed through a pharmacy [2]. The first few years of MC access were characterised by small prescription numbers amidst criticisms that the schemes were too complex, lengthy and restrictive, and products, too expensive [5, 7–9]. The last two years, however, has seen a substantial increase in prescriptions with more than 320,000 SAS-B approvals involving more than 100,000 patients and 4,600 prescribers granted at the time of writing (December 2022) [5, 10, 11].

General practitioners (GPs) are at the forefront of MC prescribing in Australia [12] and handle many patient enquiries [3, 7]. Understanding the experiences of GPs around MC in clinical practice is, therefore, of major interest. Our research team previously surveyed Australian GPs (*n* = 640) on their knowledge and attitudes towards MC in 2017, approximately one year following legalisation [7]. Results showed that most GPs were cautiously supportive of MC therapy but often felt ‘uncomfortable’ handling MC enquiries and felt poorly educated in the area. Similar outcomes have emerged from other surveys of medical practitioners in other countries [13–17].

Since our original survey, the prescribing landscape has changed considerably in Australia. The number of prescribing doctors has increased considerably and prescribing processes have been streamlined by the drug regulator and government [4]. To better understand how these developments have impacted GPs, we have conducted a new and updated survey of Australian GPs. Many of the original questions were retained, allowing us to investigate how attitudes, perceived knowledge, and concerns of GPs may have changed over time.

## Method

### Survey overview

The current survey consisted of 42-items, 21 of which were retained (or modified subtly) from our earlier 2017 survey [7]. Most of the 21 new questions probed the experiences of current MC prescribers. These were not included in the original 2017 survey as so few GPs (~ 100 in Australia) were prescribers at that time.

### Participant eligibility and recruitment

The on-line, cross-sectional survey was conducted between November 2021 – February 2022. Participants were eligible to complete the survey if they were a registered or registrar GP. All participants were required to review the Participant Information Statement and provide informed consent. Ethics approval was granted by the University of Sydney Human Research Ethics Committee on September 17, 2021 (Ref: 2021/623).

Our original (pre-COVID) GP survey was paper and pen-based and recruited audiences of GPs attending in-person, multi-topic, educational events at major Australian capital cities. These events were hosted by Healthed, an Australian provider of continuing medical education (www.healthed.com) that services a large extended network of Australian GPs [6]. During to the COVID-19 pandemic, Healthed events were moved exclusively to an on-line format meaning that our updated survey could not be conducted in person. The survey, therefore, recruited GPs accessing a 2-h, on-line, multi-topic educational event hosted by Healthed. This event and the survey were promoted to GPs on the Healthed website and through emails to 15,989 GPs from Healthed’s network of health professionals. GPs registered to attend the educational event with no cost charged for attendance. This event also included topics on lung disease, the COVID-19 pandemic and iron infusions. GPs completed the survey at their convenience up until February 2022. All participating GPs were eligible to enter a draw to win one of one hundred $100 gift vouchers.

### Survey design

The survey (see Supplementary Materials) was developed and administered using a secure, web-based platform (REDCap®12.0.7, 2022, Vanderbilt University). There were six sections and involved a total of 42-items. The survey took approximately 10-minutes to complete. The sections explored participant demographics (nine-items), MC prescribing experiences (12-items), indications for which MC was prescribed or supported (two-items), attitudes and perspectives towards MC (11-items), perceived knowledge (five-items), and concerns around THC and CBD (three-items). Branching logic was used to ensure that only current or previous MC prescribers completed the section around prescribing experience. The demographics and experience sections contained multiple choice and ‘yes-no’ style questions. Other sections involved responses across a 5-Point Likert Scale (e.g., Strongly Agree, Agree, Neutral, Disagree or Strongly Disagree). A text box to allow open-ended comments was provided at the end of the survey.

### Data management and analysis

Responses on the 5-point Likert scale questions were collapsed into the following three categories: Agree (Strongly Agree + Agree), Neutral, and Disagree (Strongly Disagree + Disagree). Clean data were synthesised using descriptive statistics (e.g., proportions, medians, ranges). MC ‘prescribers’ and ‘non-prescribers’ were compared on certain responses (demographic characteristics and perceived knowledge) using Pearson’s χ2 tests. Analyses was conducted using IBM SPSS Statistics V.24.0 (IBM, U.S.). Figures were created using GraphPad Prism V.9.3.1 (350) for Mac (GraphPad Software, La Jolla, California, USA).

Responses to open-ended questions were grouped by common theme (e.g., perceived benefits and challenges).

## Results

A total of 617 GPs initiated the survey. Those who failed to complete all six sections (*n* = 112) were removed from the analysis leaving a total of 505 participants. As the true number of GPs exposed to the advertisement of the educational event and survey through the Healthed website and email-base is unknown, the survey response rate could not be reliably calculated.

### Demographic characteristics

Participant demographics (*n* = 505) are summarised in Table 1. Most participants identified as female (59.8%) and the most common age range was ≥ 55 years (51.7%). Participants tended to be located in the state of New South Wales (33.5%), had ≥ 20 years of clinical practice experience (56.9%) and work > 30 h per week (55.2%) in metropolitan areas (64.4%). Only 8.1% of participants (*n* = 41) were registrars.

**Table 1:** Demographic characteristics (*n* = 505) of GP participants

**Characteristics**		***n* = 505**	**%**
** Gender**	Male	203	40.2
	Female	302	59.8
** Age (years)**	44 and under	129	25.5
	45–54	115	22.8
	55 and over	261	51.7
** State**	New South Wales	169	33.5
	Victoria	134	26.5
	Queensland	90	17.8
	Western Australia	50	9.9
	South Australia	38	7.5
	Tasmania	12	2.4
	Australian Capital Territory	10	2.0
	Northern Territory	2	0.4
** Years of Practice**	9 or less	114	22.6
	10–19	104	20.6
	20 or more	287	56.9
** Hours of Practice per Week**	≤ 30	226	44.7
	> 30	279	55.2
** Area Serviced**	Metropolitan Only	318	63.0
	Regional Only	140	27.7
	Remote Only	30	5.94
	Combination of metropolitan, regional and/or remote	17	3.38

### Prescribing experience

Most GP participants (85.3%, 431/505) had received at least one enquiry about MC in the preceding three months (Supplementary Table 1), with 55.4% (280/505) having received between one and four enquiries during this time. Only around half of all participants (52.3%, 264/505) felt comfortable discussing MC with patients.

Approximately one fifth of the surveyed GPs (21.8%, 110/505) had prescribed MC products during their career and were considered MC ‘prescribers.’ Of those, more than half (60.0%, 66/110) had written one to nine prescriptions in the preceding three months. Most prescriptions were new (rather than repeats) (60.9%, 67/110) and involved the SAS-B pathway (83.6%, 92/110). Prescribers varied in the number of different MC products they prescribed, from only one (32.7%, 36/110) to more than five (15.4%, 17/110).

The demographic characteristics of prescribers and non-prescribers were compared. Being a prescriber was associated with state of residence (χ^2^(7) = 16.9, *p* < 0.018) with over-representation of GPs based in Queensland. Prescribers were also over-represented (χ^2^(7) = 15.2, *p* < 0.033) among GPs with 15–29 years of clinical experience. No other significant differences were observed.

GP participants reported prescribing products that mostly contained CBD (71.8%, 79/110) and combined THC and CBD products (70.9%, 78/110). Products that mostly contained THC were less frequently prescribed (27.3%, 30/110). When asked which ‘form(s)’ of MC they had prescribed (ever), they nominated oil formulations (94.5%, 104/110); flower (also known as flos or plant material) (26.4%, 29/110); and capsules (10.9%, 12/110).

More than half of prescribers believed that cannabis should *only* be legally available for medicinal purposes (65.5%, 72/110) rather than medicinal and recreational purposes. Prescribers were equally split in their opinions on whether current access pathways were “user friendly” (50%, 55/110).

### Indications for Use

Prescribers (21.8%, 110/505) most commonly prescribed MC for chronic non-cancer pain (92.7%, 102/110), anxiety (65.5%, 72/110) and neuropathic pain (61.8%, 68/110) (Supplementary Table 2).

**Fig. 1:**
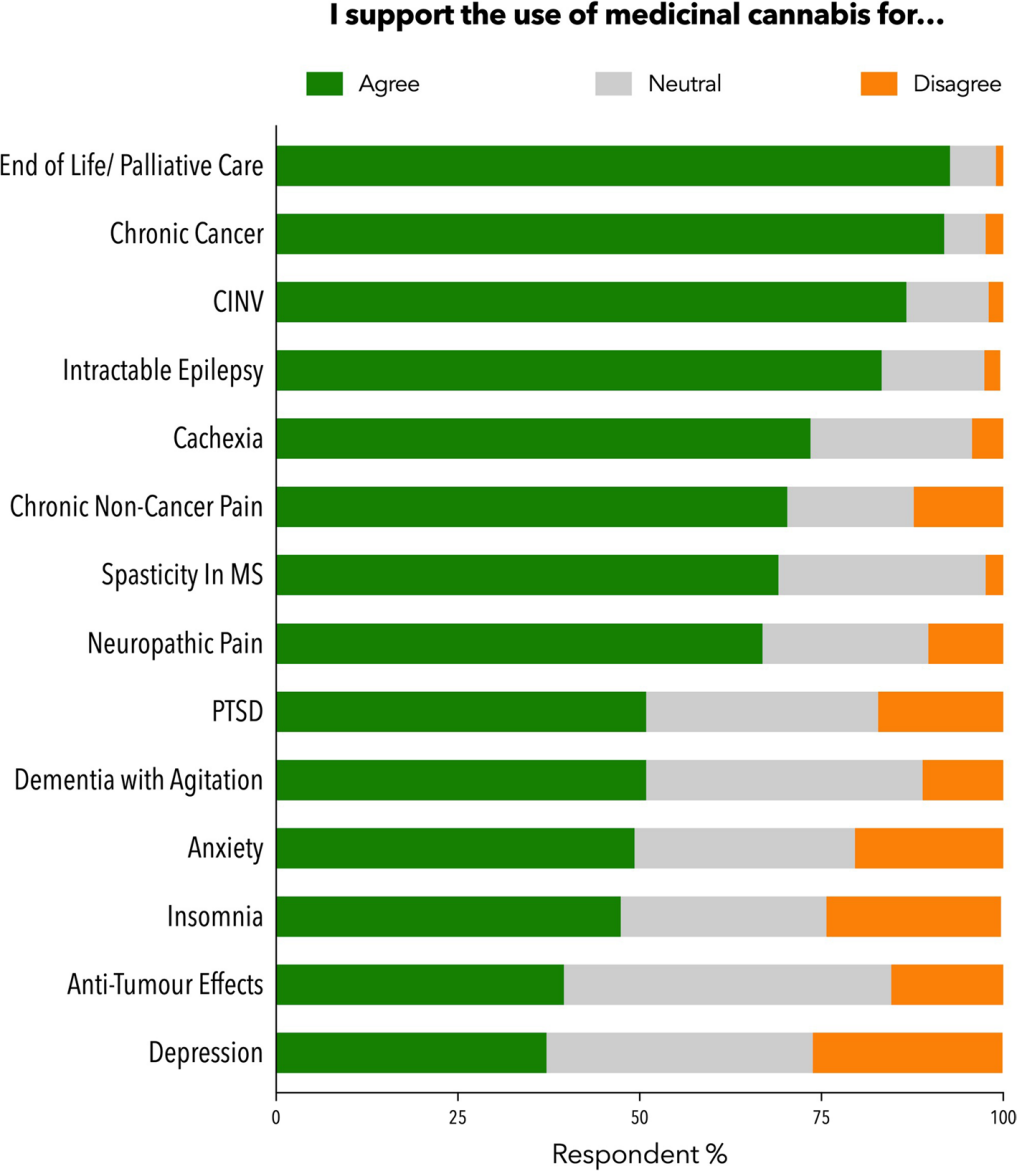
The extent to which GPs support the use of medicinal cannabis to treat certain conditions (*n* = 505). Abbreviation: CINV: chemotherapy induced nausea and vomiting; MS: multiple sclerosis; PTSD: post-traumatic stress disorder

With all GP participants considered (*n* = 505), the conditions attracting most support for MC prescribing (Fig. 1) were end of life/palliative care (92.7%, 468/505), chronic cancer pain (91.9%,464/505), chemotherapy-induced nausea and vomiting (86.7%, 438/505) and intractable epilepsy (83.8%, 423/505). Only a minority of GPs endorsed the use of MC to treat anxiety (49.3%, 249/505), insomnia (47.4%, 241/505) and depression (37.2%, 188/505).

### Attitudes

**Fig. 2:**
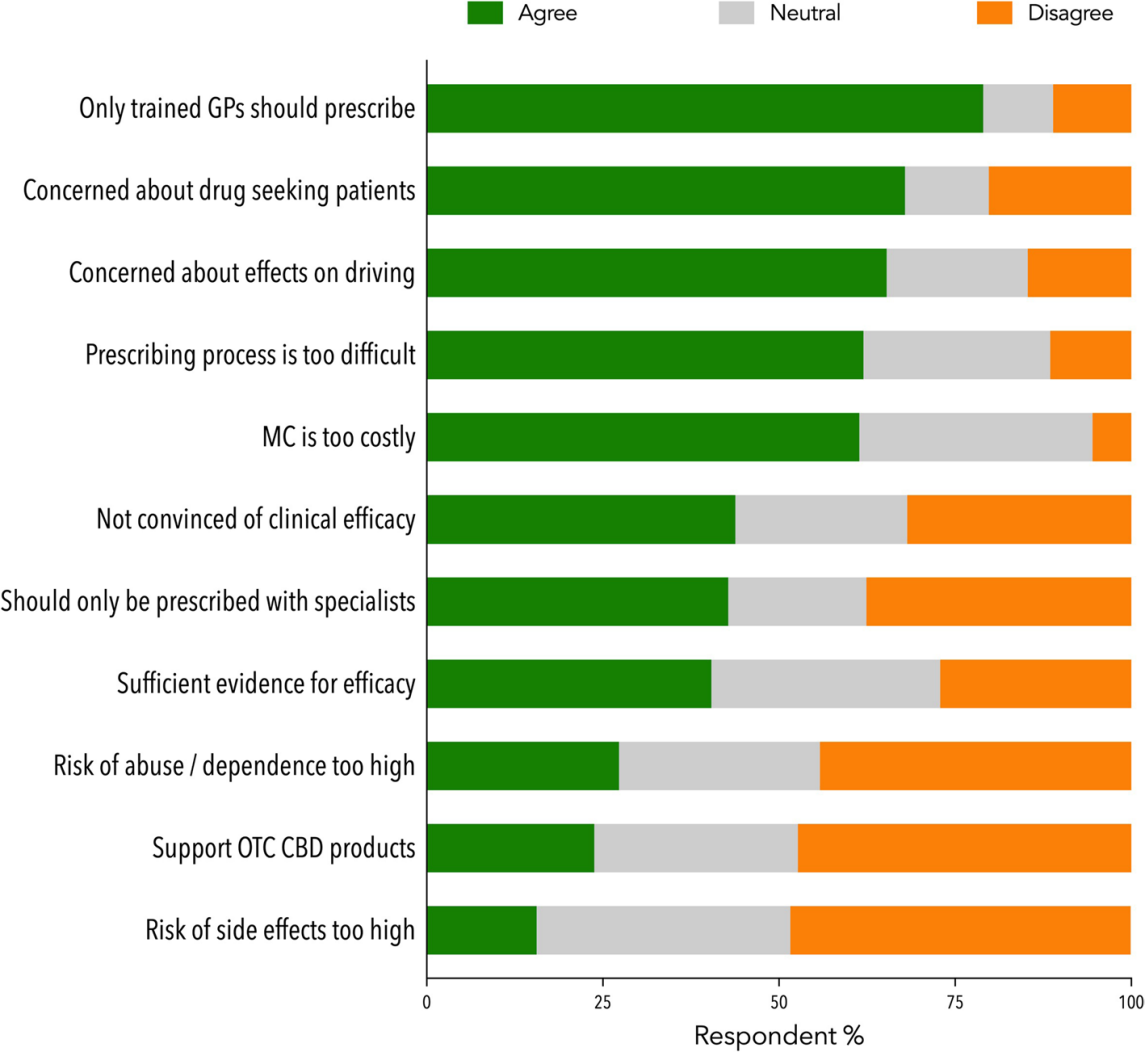
Attitudes towards, and perceptions of, MC prescribing and use (*n*=505). Abbreviations: GP: general practitioner; MC: medicinal cannabis; CBD: cannabidiol; OTC: over the counter. Refer to the Supplementary Materials for a full copy of the survey questions

More than half of the GP participants (*n* = 505) felt the prescribing processes were difficult to navigate (62.0%), and products, too costly (61.4%, Fig. 2). Many also thought GPs should have specific training to prescribe MC (79.0%). Less than half thought that GPs should only prescribe MC with specialist support (42.8%). There was little support for CBD products being made available as over the counter products in pharmacies (23.8%).

Only a minority of GP participants doubted the efficacy of MC (43.8%) while a majority expressed concerns about drug-seeking patients (67.9%) and the effects of MC on driving (65.3%). Few believed the risk of abuse and dependence (27.3%) and risk of side effects (15.6%) were “too high”.

### Perceived knowledge

**Fig. 3:**
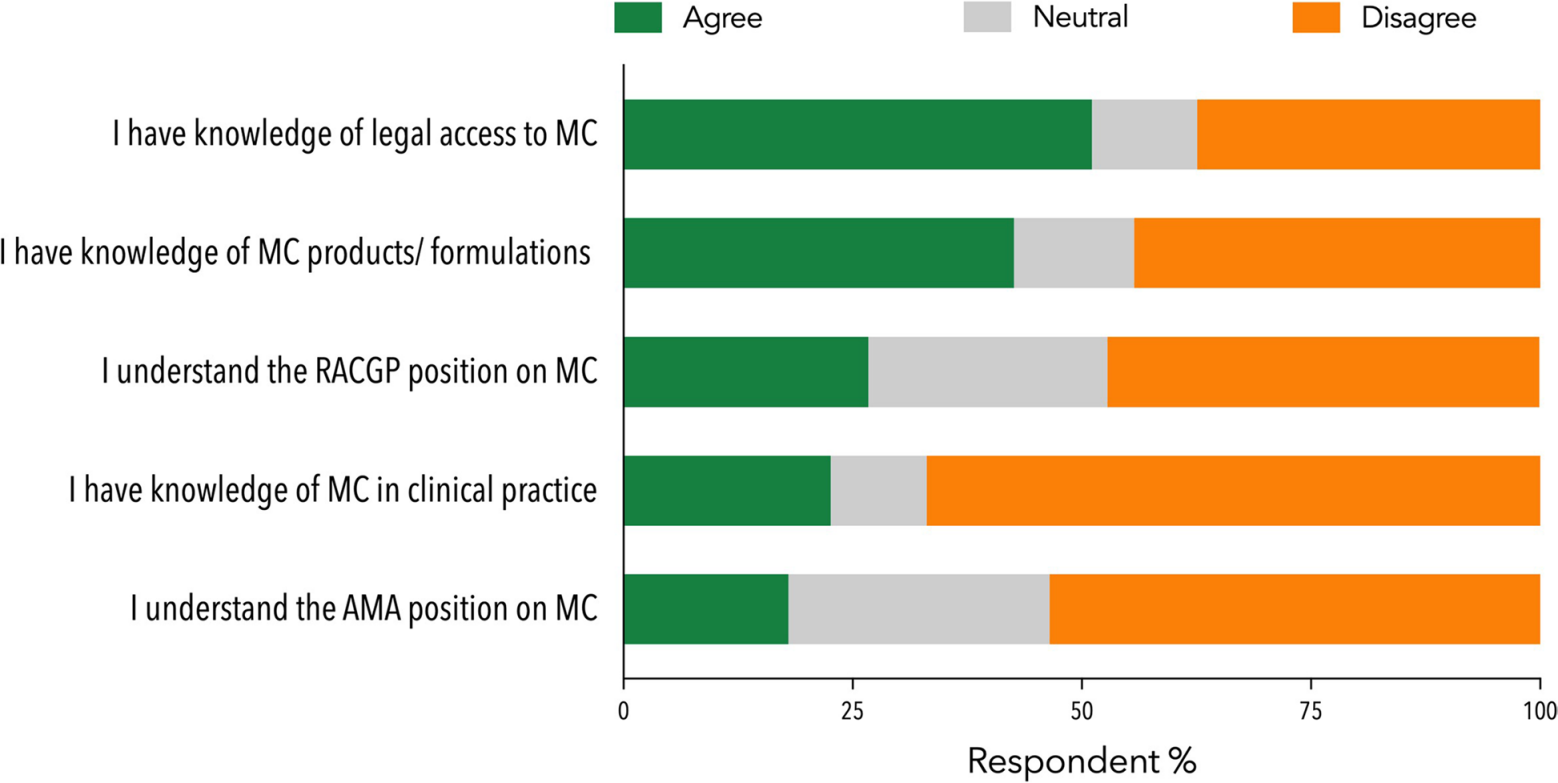
Perceived knowledge of MC as expressed by GPs (*n*=505). Abbreviations: AMA: Australian Medical Association; RACGP: Royal Australian College of General Practitioners; MC: medicinal cannabis. Refer to the Supplementary Materials for a full copy of the survey questions

Few GP participants (*n* = 505) felt they had adequate knowledge about the use of MC in clinical practice (22.6%) (Fig. 3). Only around half knew how to help patients access MC (51.1%) and less than half were aware of the products and formulations available (42.6%). Unsurprisingly, prescribers indicated higher perceived knowledge than non-prescribers (*p*’s < 0.001) (Supplementary Table 3). Few GPs were aware of the stated positions of the Australian Medical Association [18] (AMA, 18.0%) and the Royal Australian College of General Practitioners [19] (RACGP, 26.7%) around MC.

### Concerns

**Fig. 4:**
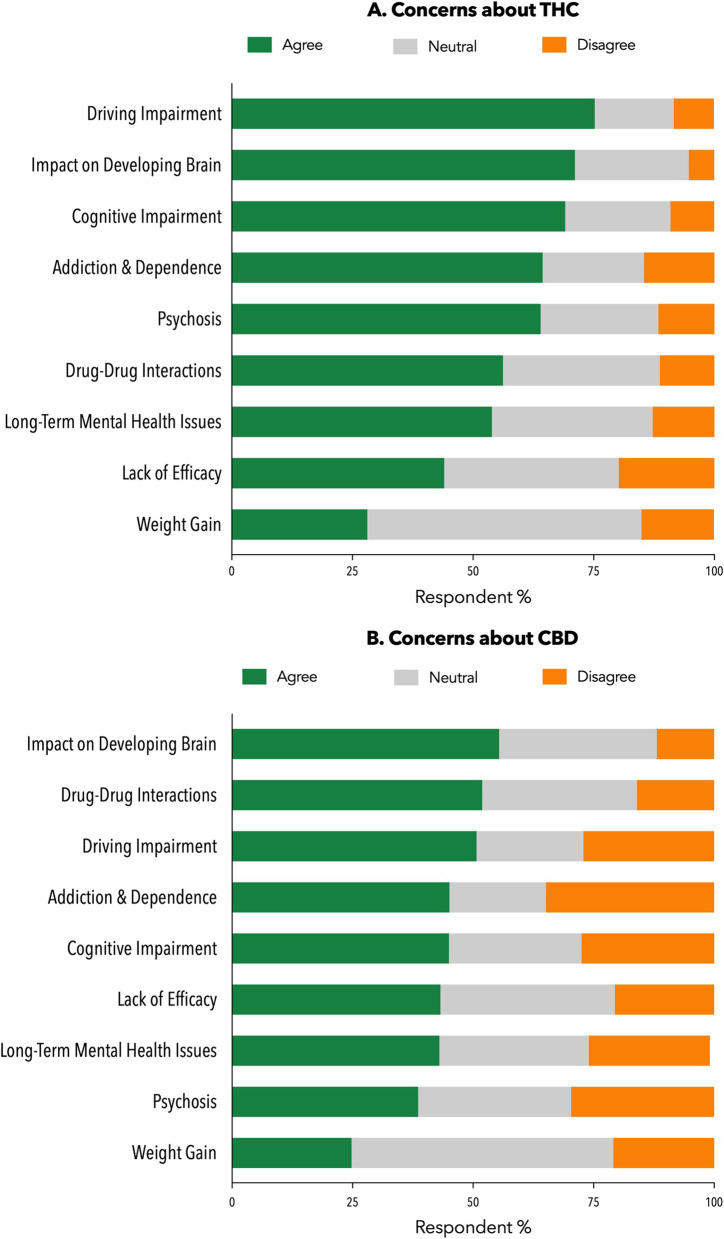
Concerns around the safety and efficacy of THC products (Panel **A**, *n*=505).Concerns around the safety and efficacy of CBD products (Panel **B**, *n*=505). Numbers show percentage of GPs (n=505) endorsing agreement, disagreement, and neutrality around specific concerns

With regard to THC-containing products (Panel A, Fig. 4), GP participants (*n* = 505) endorsed concerns around driving impairment (75.2%), impact on the developing brain (71.1%), cognitive impairment (69.1%), addiction and dependence (64.4%) and psychosis (64.0%). They were largely neutral about the concern of weight gain (56.8% neutral, 28.1% agree, 15.0% disagree).

With respect to CBD products (Panel B, Fig. 4), participants (*n* = 505) endorsed concerns about effects on the developing brain (55.4%) and possible interactions with other medications (51.9%). Driving impairment (50.7%) and addiction and dependence (45.1%) were also nominated as significant concerns with CBD even though there is negligible evidence to support such concerns.

**Fig. 5:**
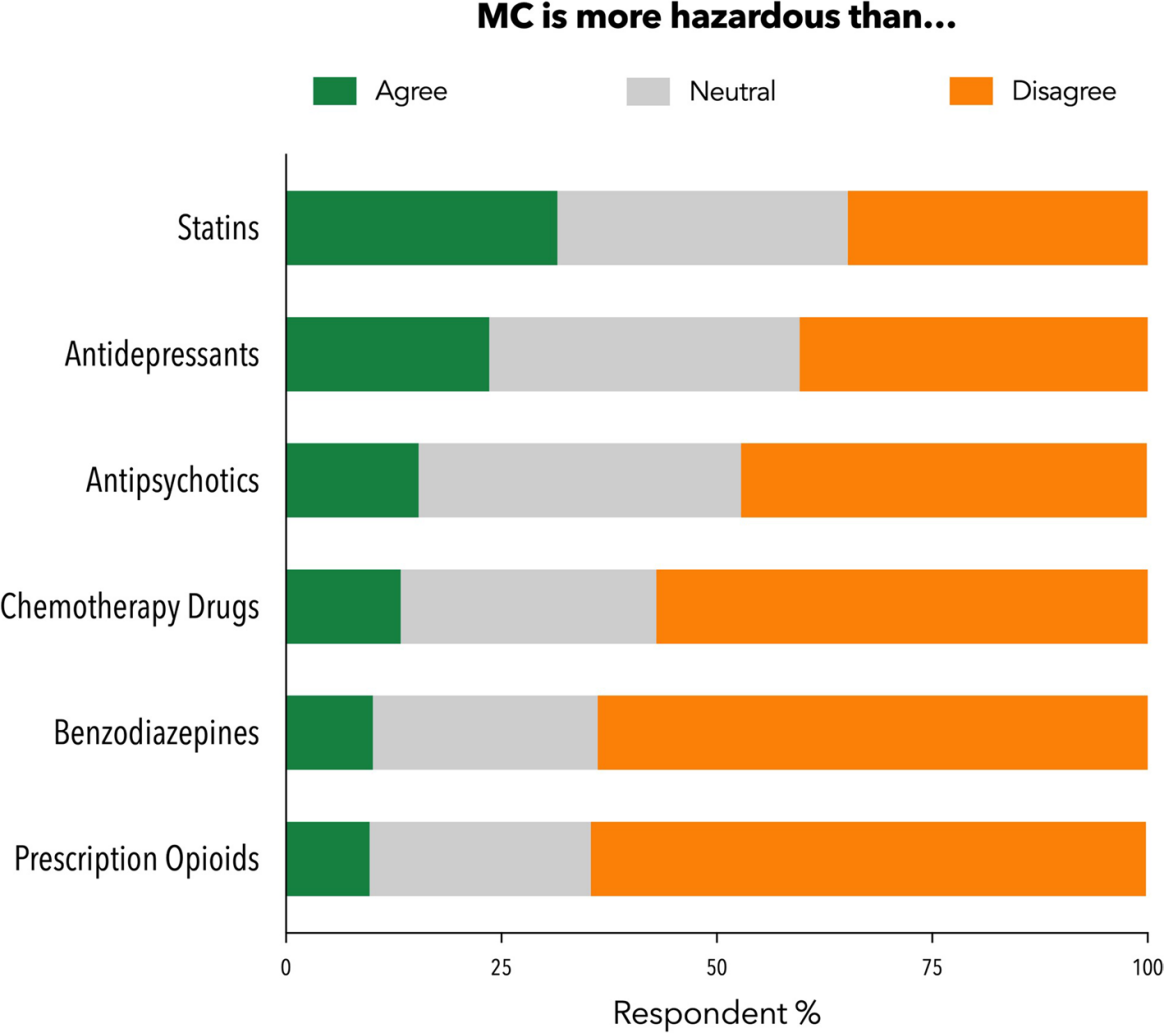
GPs tended to disagree that MC products were “more hazardous” than commonly prescribed medications (*n*=505)

Participants (*n *= 505) were asked if MC was more hazardous than commonly prescribed medications (Fig. 5). Overall, more than half of participants disagreed that MC was more hazardous than opioids (64.4%), benzodiazepines (63.8%) and chemotherapy drugs (57.0%). Participants also tended to disagree that MC was more hazardous than antipsychotics (47.1%), antidepressants (40.4%) and statins (34.9%) although a sizeable proportion endorsed neutrality around these latter comparisons.

### Open ended comments

Open-ended comments provided by participants (*n* = 86) commonly centred around the need for knowledge development (29%, 25/86) in this area. Cost issues (14.0%, 12/86), the importance of MC and its availability as a treatment option for chronic conditions (11.6%, 10/86), and the difficult or complicated prescribing process (8.1%, 7/86), were also noted by multiple participants.

## Discussion

This study explored the current knowledge, experiences, and attitudes of Australian GPs towards MC, and provides key insights into this emerging field subsequent to our original survey of five years ago [7]. This survey is reasonably comprehensive, with 42-items scoping numerous aspects of MC that are relevant and specific to general practice. Overall, most GPs had received MC enquiries from patients. Yet only a minority felt comfortable managing these enquiries. Only around 21% of the cohort were MC ‘prescribers’, around the same proportion (22%) of the overall cohort reporting adequate self-perceived knowledge around MC in clinical practice. Indeed, many GPs felt they had inadequate knowledge of MC products, MC access pathways and optimal clinical practice. A need for further education in this area was also endorsed in the open-ended comments.

A disproportionate number of MC prescribers resided in Queensland, in agreement with recent government data on prescriber location for SAS-B approvals [5]. Overall, however, GP demographics in the current survey were strikingly similar to our original 2017 survey [7]. More GPs had received recent MC enquiries than in our original survey (85.3% vs. 61.5% respectively in past three months). This observation is consistent with the recent and considerable rise in MC prescribing [2, 10] likely driven by recent initiatives aimed at improving access pathways, as well as the growing acceptance of MC from health professionals [10, 20, 21]. The mental health burden of the COVID-19 pandemic has also been suggested as a contributing factor for the demand for MC products over the past two years [10].

Interestingly, however, GP comfort levels with managing MC enquiries remain low and unchanged since the original survey despite the increase in patient enquiries. Low comfort levels around managing and prescribing cannabis-based therapy are in line with other surveys of physicians [13–17]. Australian GPs currently select from > 240 distinct MC products to treat more than 120 distinct medical conditions and often prescribe in the absence of high-quality supporting evidence of efficacy [3, 21, 22]. The specialised prescribing process and costly nature of MC products may also present hurdles in GP discussions with patients [7, 9, 23]. Most GP participants endorsed these two issues as ongoing problems. Formal education around MC has been suggested to improved comfort levels [14, 15, 24].

The greatest support for the use of MC was in terminal and/or often difficult-to-treat medical conditions such as palliative care, chronic cancer pain and CINV, more so than our original survey (Supplementary Table S4) [7] and similar to other international studies [25–27]. MC is often seen as a therapy of last resort, where conventional treatment options have been exhausted [10, 15, 25, 27, 28]. Furthermore, the evidence around the use of cannabis-based medicine in these indications has been well-reviewed and summarised in a variety of comprehensive guidance documents by organisations such as the TGA [2, 3, 21].

The extent of participating GPs support for the use of MC in anxiety, depression and insomnia was not well-endorsed relative to other indications, perhaps reflecting the limited evidence for MC efficacy in these conditions [29, 30]. Additional factors here may be the availability of relatively safe and effective conventional therapies for these conditions, as well as concerns around the impact of THC use on mental health [16, 27, 31]. When compared to the original survey however, participating GPs demonstrated increased support for the use of MC in most health conditions compared to the original survey (Supplementary Table 4) [7] and this included mental health conditions such as anxiety and PTSD as well as insomnia and chronic pain. This agrees with TGA data showing chronic pain, anxiety and sleep disturbance as the leading indications for SAS-B approvals [5].

Participating GPs who were MC prescribers had prescribed MC most often for chronic pain and anxiety (Supplementary Table 2). However, prescribing for these indications is supported by relatively sparse evidence [32–35]. Indeed, participating GPs were clearly aware of the limited evidence for clinical efficacy of MC, as has been noted in previous surveys [13, 16, 17, 31]. Widespread prescribing of MC in the absence of evidence suggests a patient-driven rather GP-driven process, or possibly that clinical efficacy is readily apparent to GPs but has yet to be properly captured by published clinical trials.

Concerns endorsed by GPs around THC included driving impairment, addiction and dependence; and are legitimate given current evidence [22, 36]. Some GPs expressed concern around THC causing weight gain; however, regular cannabis users tend to have a leaner phenotype than non-users [37, 38]. Legitimate concerns were expressed around drug-drug interactions with CBD [22]. However, concerns expressed around CBD causing driving and cognitive impairment are unfounded [39, 40]. Similarly, concerns existed around addiction and psychosis, yet CBD has shown some promise as a *treatment* for these conditions [41, 42]. Concerns around MC are therefore not always based on the most current evidence. The lack of endorsement for availability of low dose CBD products in pharmacies may reflect misconceptions around the safety of such products by GPs.

Despite safety concerns, few GPs believed the risk of side effects were “too high” (15.6% in the current survey vs. 19.8% in the original survey). Notably, GPs rated prescription opioids, benzodiazepines, and chemotherapy drugs as more hazardous than MC products, to a greater extent than the original survey [7]. This indicates increasing acknowledgement of the safety of cannabis-based medicines as clinical experience with MC grows.

The low perceived knowledge of GPs around MC, particularly amongst non-prescribers, is perhaps the most striking outcome of the survey and was reiterated in open-ended comments. Notably, most GPs (79%) endorsed the need for compulsory, MC-specific training for prescribers, a belief that has been maintained since the original survey (78.6%). Low knowledge has been highlighted repeatedly in previous surveys of physician around MC [13, 15–17, 26]. It is essential that GPs are supported in developing a sound knowledge around MC, regardless of whether they prescribe MC products or not. Professional organisations will play a leading role in educational initiatives in this area. Knowledge drives optimal patient care [7], and lack thereof may contribute to inadequate provision of support including missed or delayed treatment opportunities [43]. This should be a priority given the ongoing surge in demand for MC therapy.

There are around 4600 MC prescribers in Australia [5] of whom approximately 80% are thought to be GPs (i.e., around 3680) [12]. With a total of 31,000 GPs in Australia [44] MC prescribers were perhaps over-represented in our cohort (21%; 110/505) compared to the overall GP population (12%, 3680/31,000). The 505 GPs included in this study is only a small proportion of the total Australian population of ~ 31,000 GPs [45] such that issues of sample representativeness are worthy of consideration. Government data show Australian GPs to be 41.0% identifying as female, 40.0% aged ≥ 55 years and 32.1% located in NSW [45] compared to 59.8% identifying as female, 51.7% aged ≥ 55 years and 33.5% located in NSW respectively in the current study. Other limitations include recruitment being limited to GPs participating in continuing education and use of an on-line recruitment strategy due to the COVID-19 pandemic that may have biased the cohort towards more technologically adept GPs.

## Conclusion

There is accelerating demand for MC products in Australia, in line with global trends [23]. GPs are well positioned to assist in the safe and efficacious use of cannabis-based medicines in the community. Training around MC is required to address the long-standing concerns of GPs around knowledge and confidence in this area. Findings from this study can be used to develop educational initiatives and promote best practice. Australia has the potential to act as exemplar for other countries in guiding high quality MC prescribing and product utilisation in a government-regulated model. Many countries, such as the United Kingdom, face similar access challenges as those of Australia but are yet to utilise GPs as independent MC prescribers, limiting MC prescribing approvals to specialists only [46, 47].

## Supplementary Information

**Additional file 1: Table S1. **Experiences around Patient Enquiries and Prescribing (*n*=505). **Table S2.** Indications that prescribers have treated with medicinal cannabis (*n*=110, valid percentage). **Table S3.** χ2 Test Values for each of the Knowledge questions in the GP Survey. **Table S4.** Differences in support for common MC indications between the current survey and the original survey (Karanges et al., 2018).

## Data Availability

The datasets generated during and/or analysed during the current study are available from the corresponding author on reasonable request.

## Abbreviations

MC: Medicinal cannabis
GP: General practitioners
THC: Tetrahydrocannabinol
CBD: Cannabidiol
SAS: Special Access Schemes
AP: Authorised Prescriber
