# Supplementary material for: Knowledge, experiences, and attitudes of Australian General Practitioners towards medicinal cannabis: a 2021–2022 survey
Source: BMC Prim Care. 2022 Dec 19;23:330. doi: 10.1186/s12875-022-01946-x (PMC9760535; doi:10.1186/s12875-022-01946-x)
Supplement: Supplementary file 1 — Additional file 1: Table S1. Experiences around Patient Enquiries and Prescribing (n=505). Table S2. Indications that prescribers have treated with medicinal cannabis (n=110, valid percentage). Table S3. χ2 Test Values for each of the Knowledge questions in the GP Survey. Table S4. Differences in support for common MC indications between the current survey and the original survey (Karanges et al., 2018). [file 12875_2022_1946_MOESM1_ESM.pdf]

Supplementary Material

Table S1. Experiences around Patient Enquiries and Prescribing (n=505)

Experience		n=505	%
**MC Enquiries in past 3 Months**	Nil	74	14.7
	1-4	280	55.4
	5-9	103	20.4
	10-19	30	5.9
	20 or more	18	3.6
**Comfortable Discussing Enquiries**	Yes	264	52.3
	No	241	47.7
**MC Prescriber**	Yes	110	21.8
	No	395	78.2
**Prescriptions in Past 3 Months**	0	410	81.2
	1-9	66	13.1
	10-19	15	3.0
	20-49	11	2.2
	50 or more	3	0.6
**Type of Prescriptions**	Repeat	43	8.5
	New Prescription	67	13.3
**Product Type (THC and/or CBD)**	THC Dominant	30	5.9
	CBD Dominant	79	15.6
	THC and CBD Combination	78	15.4
**Distinct Products Prescribed**	1	36	7.1
	2	21	4.2
	3	24	4.8
	4	12	2.4
	More than 5	17	3.4
**Formulation Types Prescribed**	Capsules	12	2.4
	Oil	104	20.6
	Sprays	9	1.8
	Flower (flos, plant material)	29	5.7
	Other oral products e.g., tablets, lozenges	3	0.6
**MC Prescribing Pathway Used**	Special-Access Scheme B	92	18.2
	Authorised Prescriber Scheme	38	7.5
**Are Pathways User Friendly?**	Yes	55	10.9
	No	55	10.9
**Support for Legal cannabis**	Medicinal	72	14.3
	Recreational	0	0
	Both	38	7.5

MC: Medicinal cannabis.

Table S2. Indications that prescribers have treated with medicinal cannabis (n=110, valid percentage)

MC Prescribed for Condition	N=110	%
**Chronic Non-Cancer Pain**	102	92.7
**Anxiety**	72	65.5
**Neuropathic Pain**	68	61.8
**Insomnia**	51	46.4
**PTSD**	43	39.0
**Chronic Cancer Pain**	42	38.2
**Depression**	35	32.0
**End of Life/Palliative Care**	28	25.5
**Chemotherapy-Induced Nausea and Vomiting**	19	17.3
**Spasticity in Multiple Sclerosis**	16	14.5
**Other**	13	11.8
**Dementia Patients with Agitation**	10	9.1
**Intractable Epilepsy**	7	6.4
**Cachexia Associated with Severe Illness**	6	5.5
**Anti-Tumour Effects**	3	2.7

MC: Medicinal cannabis; PTSD: Post-traumatic stress disorder.

Table S3. χ^2 Test Values for each of the Knowledge questions in the GP Survey

Knowledge Question Number	Topic	χ^2 Test Value
**Questions 1**	Knowledge	($\chi^2(2)$ =120.5, p<0.001)
**Questions 2**	AMA	($\chi^2(2)$ =33.6, p<0.001)
**Questions 3**	RACGP	($\chi^2(2)$ =35.8, p<0.001)
**Questions 4**	MC formulations	($\chi^2(2)$ =88.8, p<0.001)
**Questions 5**	Helping patients	($\chi^2(2)$ =83.6, p<0.001)

Table S4. Differences in support for common MC indications between the current survey and the original survey (Karanges et al., 2018)

Indication	% Support Current Survey (N=505)	% Support Original Survey (N=640)	% Difference
**Anxiety**	49.3	13	36.3
**Insomnia**	47.4	13.2	34.2
**PTSD**	50.9	19	31.9
**Chronic Non-Cancer Pain**	70.3	39.1	31.2
**Neuropathic Pain**	66.9	38.3	28.6
**Depression**	37.2	9.4	27.8
**CINV**	86.7	64.7	22
**Cachexia**	73.5	56.7	16.8
**End of Life/Palliative Care**	92.7	78.8	13.9
**Intractable Epilepsy**	83.3	70.3	13
**Chronic Cancer Pain**	91.9	80.2	11.7
**Dementia with Agitation**	50.9	40.2	10.7
**Spasticity in MS**	69.1	61.2	7.9

Section 1 - Anonymous demographic information

Question	Options
Age category	◯ Under 24<br>◯ 25-34<br>◯ 35-44<br>◯ 45-54<br>◯ 55-64<br>◯ Over 65
Gender	◯ Male<br>◯ Female<br>◯ Other
State/Territory of your practice	◯ NSW<br>◯ VIC<br>◯ QLD<br>◯ SA<br>◯ TAS<br>◯ WA<br>◯ ACT<br>◯ NT
Are you a GP registrar?	◯ Yes<br>◯ No
Years in General Practice	◯ Less than 1<br>◯ 1 - 4<br>◯ 5 - 9<br>◯ 10 - 14<br>◯ 15 - 19<br>◯ 20 - 24<br>◯ 25 - 29<br>◯ More than 30
Average hours spent in clinical practice per week	◯ Less than 10<br>◯ 11 - 20<br>◯ 21 - 30<br>◯ 31 - 40<br>◯ More than 40
How many GPs work at your practice?	◯ 1<br>◯ 2 - 4<br>◯ 5 - 9<br>◯ 10 - 20<br>◯ More than 20
Geographical area serviced (Select all that apply)	☐ Metro<br>☐ Regional<br>☐ Remote
What is the post code of the practice where you work? (Optional)	__________________________

Section 2 - Medicinal cannabis experience

In the past 3 months, how many of your patients have enquired about medicinal cannabis products?	☐ 0<br>☐ 1-4<br>☐ 5-9<br>☐ 10-19<br>☐ 20+
Do you feel comfortable discussing medicinal cannabis as a treatment option with your patients?	◯ Yes<br>◯ No
Have you prescribed medicinal cannabis?	◯ Yes<br>◯ No
In the past 3 months, how many medicinal cannabis prescriptions have you provided?	◯ 0<br>◯ 1-9<br>◯ 10-19<br>◯ 20-49<br>◯ 50-99<br>◯ 100-149<br>◯ 150+
Are the majority of your medicinal cannabis prescriptions repeats or new prescriptions?	◯ Repeats<br>◯ New Prescriptions
What type of medicinal cannabis products have you prescribed? (Tick all that apply)	☐ THC dominant (Schedule 8)<br>☐ CBD dominant (Schedule 4)<br>☐ THC & CBD combination (Schedule 8)
How many distinct medicinal cannabis products do you currently prescribe?	◯ 1<br>◯ 2<br>◯ 3<br>◯ 4<br>◯ More than 5
What formulations of medicinal cannabis have you prescribed? (Tick all that apply)	☐ Capsules<br>☐ Oil<br>☐ Sprays<br>☐ Flower (Flos, plant material)<br>☐ Other oral products e.g., tablets, lozenges
How have you prescribed medicinal cannabis products? (Tick all that apply)	☐ Special-Access Scheme B<br>☐ Authorised Prescriber Scheme
Do you believe the TGA approval process for medicinal cannabis is doctor-friendly?	◯ Yes<br>◯ No
Do you believe cannabinoids should be legally available for the following purposes?	◯ Medicinal<br>◯ Recreational<br>◯ Both

I have prescribed medicinal cannabis products for the following conditions (tick all that apply)	☐ Chronic cancer pain<br>☐ Chronic non-cancer pain<br>☐ Neuropathic pain<br>☐ Intractable epilepsy<br>☐ End of life/Palliative care<br>☐ Chemotheraphy-induced nausea and vomiting<br>☐ Cachexia associated with severe illness<br>☐ Anti-tumour effects<br>☐ Spasticity in Multiple Sclerosis<br>☐ Dementia patients with agitation<br>☐ Insomnia<br>☐ PTSD<br>☐ Anxiety<br>☐ Depression<br>☐ Other (please specify)
Other, please specify	______________________________

Please indicate how much you agree or disagree with each of the following statements

	Strongly Agree	Slightly Agree	Neutral	Slightly Disagree	Strongly Disagree
I know how to help patients legally access medicinal cannabis	◯	◯	◯	◯	◯
I am aware of the different medicinal cannabis products and formulations currently available	◯	◯	◯	◯	◯
I understand the current RACGP position on medicinal cannabis	◯	◯	◯	◯	◯
I understand the current AMA position on medicinal cannabis	◯	◯	◯	◯	◯
I am supportive of CBD products available over the counter in pharmacies	◯	◯	◯	◯	◯
Medicinal cannabis should only be prescribed by GPs in conjunction with specialists	◯	◯	◯	◯	◯
Only GPs who have undergone specific training and credentialing should be allowed to prescribe medicinal cannabis	◯	◯	◯	◯	◯
There is sufficient scientific evidence on the efficacy of medicinal cannabis	◯	◯	◯	◯	◯

When considering prescribing medicinal cannabis

	Strongly agree	Slightly agree	Neutral	Slightly disagree	Strongly disagree
The risk of side effects is too high	○	○	○	○	○
The risk of abuse and dependence is too high	○	○	○	○	○
I am concerned about the effects on driving	○	○	○	○	○
I am concerned about drug seeking patients	○	○	○	○	○
I do not have enough knowledge of its application in clinical practice	○	○	○	○	○
The prescribing process is too difficult	○	○	○	○	○
I am not convinced of the clinical efficacy	○	○	○	○	○
It is too costly	○	○	○	○	○
There is sufficient scientific evidence on the efficacy of medicinal cannabis	○	○	○	○	○

I support the use of medicinal cannabis products in patients with

	Strongly agree	Slightly agree	Neutral	Slightly disagree	Strongly disagree
Chronic cancer pain					
Chronic non-cancer pain					
Neuropathic pain					
Intractable epilepsy					
End of life/Palliative care					
Chemotheraphy-induced nausea and vomiting					
Cachexia associated with severe illness					
Anti-tumour effects					
Spasticity in Multiple Sclerosis					
Dementia patients with agitation					
Insomnia					
PTSD					
Anxiety					
Depression					

The major concerns with THC containing products include:

	Strongly agree	Slightly agree	Neutral	Slightly disagree	Strongly disagree
Addiction and dependence	○	○	○	○	○
Cognitive impairment	○	○	○	○	○
Driving impairment	○	○	○	○	○
Weight gain	○	○	○	○	○
Psychosis	○	○	○	○	○
Other long-term mental health issues	○	○	○	○	○
Interactions with other medications	○	○	○	○	○
Impact on the developing brain	○	○	○	○	○
Lack of Efficacy	○	○	○	○	○

The major concerns with CBD containing products include:

	Strongly agree	Slightly agree	Neutral	Slightly disagree	Strongly disagree
Addiction and dependence	○	○	○	○	○
Cognitive impairment	○	○	○	○	○
Driving impairment	○	○	○	○	○
Weight gain	○	○	○	○	○
Psychosis	○	○	○	○	○
Other long-term mental health issues	○	○	○	○	○
Interactions with other medications	○	○	○	○	○
Impact on the developing brain	○	○	○	○	○
Lack of Efficacy	○	○	○	○	○

Medicinal cannabis is generally more hazardous than:

	Strongly agree	Slightly agree	Neutral	Slightly disagree	Strongly disagree
Prescription opioids	○	○	○	○	○
Benzodiazepines	○	○	○	○	○
Antipsychotics	○	○	○	○	○
Statins	○	○	○	○	○
Chemotherapy drugs	○	○	○	○	○
Antidepressants	○	○	○	○	○

Please provide any further comments, questions or opinions about medicinal cannabis in Australia

____
